# Novel biomarkers for the prediction of COVID-19 progression a retrospective, multi-center cohort study

**DOI:** 10.1080/21505594.2020.1840108

**Published:** 2020-11-11

**Authors:** Yalan Yu, Tao Liu, Liang Shao, Xinyi Li, Colin K. He, Muhammad Jamal, Yi Luo, Yingying Wang, Yanan Liu, Yufeng Shang, Yunbao Pan, Xinghuan Wang, Fuling Zhou

**Affiliations:** a Department of Hematology, Zhongnan Hospital of Wuhan University, Wuhan, China; b Department of Urology, Zhongnan Hospital of Wuhan University, Wuhan, China; c Department of Anesthesiology, Zhongnan Hospital of Wuhan University, Wuhan, China; d Orient Health Care, Stego Tech LLC, King of Prussia, PA, USA; e Department of Immunology, School of Basic Medical Science, Wuhan University, Wuhan, China; f Department of Laboratory Medicine, Zhongnan Hospital of Wuhan University, Wuhan, China; g Evidence-Based and Translational Medicine, Zhongnan Hospital of Wuhan University, Wuhan, China

**Keywords:** COVID-19, serum amyloid A protein, disease progression, risk factor, predictor, biomarker

## Abstract

A pandemic designated as Coronavirus Disease 2019 (COVID-19), caused by severe acute respiratory syndrome coronavirus 2 (SARS-CoV-2) is spreading worldwide. Up to date, there is no efficient biomarker for the timely prediction of the disease progression in patients. To analyze the inflammatory profiles of COVID-19 patients and demonstrate their implications for the illness progression of COVID-19. Retrospective analysis of 3,265 confirmed COVID-19 cases hospitalized between 10 January 2020, and 26 March 2020 in three medical centers in Wuhan, China. Patients were diagnosed as COVID-19 and hospitalized in Leishenshan Hospital, Zhongnan Hospital of Wuhan University and The Seventh Hospital of Wuhan, China. Univariable and multivariable logistic regression models were used to determine the possible risk factors for disease progression. Moreover, cutoff values, the sensitivity and specificity of inflammatory parameters for disease progression were determined by MedCalc Version 19.2.0. Age (95%CI, 1.017 to 1.048; *P* < 0.001), serum amyloid A protein (SAA) (95%CI, 1.216 to 1.396; *P* < 0.001) and erythrocyte sedimentation rate (ESR) (95%CI, 1.006 to 1.045; *P* < 0.001) were likely the risk factors for the disease progression. The Area under the curve (AUC) of SAA for the progression of COVID-19 was 0.923, with the best predictive cutoff value of SAA of 12.4 mg/L, with a sensitivity of 83.9% and a specificity of 97.67%. SAA-containing parameters are novel promising ones for predicting disease progression in COVID-19.

## Introduction

The outbreak of COVID-19, caused by SARS-CoV-2 has influenced the whole world [1–5]. By 26 July 2020, 86,967 confirmed cases, as well as 4,659 death of COVID-19, had been reported in China. Approximately 16,036,072 confirmed cases and 641,496 deaths have been reported outside of China [6]. The Chinese Center for Disease Control and Prevention has reported that the basic reproductive number of SARS-CoV-2 in China is 2.2, indicating that one COVID-19 patient can cause infection of 2 ~ 3 other individuals [7,8]. The most common initial clinical manifestations of COVID-19 are fever, dry cough, fatigue, and shortness of breath. The majority of COVID-19 cases are asymptomatic, mild or ordinary, whereas one-fifth of cases are severe or critically ill cases. The estimated overall mortality rate is 2～3% in China, but half of the critically ill patients in Wuhan finally died due to life-threatening complications [9,10].

Recently, clinical practitioners are focusing on two major questions. When does the disease progress from mild to severe? And, in this regard, are there any laboratory parameters that can be used as an alert to the front-line clinicians? Herein, we retrospectively studied 3,265 hospitalized patients with COVID-19 in three medical centers in Wuhan and investigated the changes in several inflammatory parameters during the progression of the disease. Our study aims to find an informative marker to predict the progression of patients with COVID-19 from mild to more severe stages.

## Methods

### Study design

A total of 3,265 patients with laboratory-confirmed COVID-19, admitted to Leishenshan Hospital, Zhongnan Hospital of Wuhan University and the Seventh Hospital of Wuhan during the period from 10 January 2020, to 26 March 2020, were included in this multi-centered, retrospective cohort study. These three hospitals were the designated hospitals by the government for hospitalizing COVID-19 patients in Wuhan. All participants met the criteria for the clinical diagnosis based on The National Health Commission of China (NHCC) Guidelines (7th Edition) on COVID-19. Briefly, patients with two of the following clinical symptoms plus one epidemiological risk were diagnosed as suspected COVID-19. (1) Clinical manifestations: fever, dry cough, shortness of breath, imaging feature of pneumonia, as well as low or normal white blood cell (WBC) or low lymphocyte count in the peripheral blood; (2) Epidemiological risk factors: a history of travel to Wuhan or a resident history in Wuhan or the neighboring regions within two weeks; or being exposed to confirmed COVID-19 patients; or having a close contact with the patients with respiratory symptoms or patients from the regions containing confirmed COVID-19 cases; or clustering cases. The suspected patients would be then received the laryngeal swabs test using SARS-CoV-2 PCR Nucleic Acid Diagnostic Kit according to the manufacturer’s guidance.

According to the NHCC Guidelines (7th Edition), COVID-19 patients at the time of confirmed diagnosis of COVID-19 were stratified as follows: mild (i.e. having mild clinical symptoms without imaging feature of pneumonia), ordinary (i.e. having clinical symptoms, such as fever, cough, as well as imaging feature of pneumonia), severe (i.e. having dyspnea, respiratory frequency ≥ 30/min, blood oxygen saturation ≤ 93%, partial pressure of arterial oxygen to fraction of inspired oxygen ratio < 300, and/or lung infiltrates > 50% within 24 to 48 hours), and critically ill cases (i.e. having respiratory failure, septic shock, and/or multiple organ dysfunction or failure).

This study was conducted according to the principles of Helsinki and approved by the Ethics Committee of Zhongnan Hospital of Wuhan University (No.2020063). Data were collected and independently reviewed by three physicians. Due to the urgent need for the understanding of this emerging infectious disease, the requirements for written informed consent from the participants were waived.

### SARS-CoV-2 nucleic acid test

All samples were processed at the Department of Laboratory Medicine of Leishenshan Hospital and Zhongnan Hospital of Wuhan University. All patients were tested for SARS-CoV-2 nucleic acid by the use of quantitative real-time polymerase chain reaction (qRT-PCR) on samples from the respiratory tract. Laryngeal swab samples were collected for extracting RNAs from participants suspicious of SARS-CoV-2 infection. After sample collection, the laryngeal swabs were placed into a tube containing 150 μL of virus preservation solution, and total RNA was extracted within two hours by using the respiratory sample RNA isolation kit (Zhongzhi, Wuhan, China). In detail, cell lysates were transferred into a collection tube, followed by a vortex for 10 seconds. After stewing at room temperature for 10 minutes, it was centrifuged at 1000 rpm/min for 5 minutes. Then the suspension was collected and used for real-time RT-PCR. Two target genes, including an open reading frame 1ab (ORF1ab) and nucleocapsid protein (N) were simultaneously amplified. Target 1 (ORF1ab): forward primer CCCTGTGGGTTTTACACTTAA; reverse primer ACGATTGTGCATCAGCTGA; and the probe 5ʹ-VIC-CCGTCTGCGGTATGTGGAAAGGTTATGG-BHQ1-3ʹ. Target 2 (N): forward primer GGGGAACTTCTCCTGCTAGAAT; reverse primer CAGACATTTTGCTCTCAAGCTG; and the probe 5ʹ-FAM- TTGCTGCTGCTTGACAGATT-TAMRA-3ʹ. The real-time RT-PCR assay was performed using a SARS-CoV-2 nucleic acid detection kit according to the protocol (Shanghai Bio-germ Medical Technology Co Ltd). The real-time PCR assay was performed under the following conditions: incubation at 50℃ for 15 minutes and 95℃ for an additional 5 minutes, denaturation at 94℃ for 15 seconds, as well as extension and fluorescence signaling at 55℃ for 45 seconds. According to the recommendation by the National Institute for Viral Disease Control and Prevention (China), positive results were defined as Ct-value < 37, whereas negative results were Ct-value ≥ 40.

### Laboratory tests for inflammatory markers

The common inflammatory parameters in clinic, C-reactive protein (CRP; Sekisui Medical Co., Tokyo, Japan) and serum amyloid A (SAA; Purebio Biotech Co., Ningbo, China) were tested on an Olympus 5800 analyzer (Beckman Coulter) using the latex-enhanced immunoturbidimetric assay. Interleukin-6 (IL-6) was detected using the automatic electrochemiluminescence immunoassay (ECLIA) system (Cobas e601, Roche), and procalcitonin (PCT) was tested on an automatic immunoassay analyzer (VIDAS, Biomerieux, France) using the enzyme-linked fluorescence analysis (ELFA).

We also measured lymphocyte subsets in samples of EDTA anti-coagulated peripheral blood from patients with COVID-19 on admission using multiple-color flow cytometry. The cells were analyzed on a BD FACS Canto Ⅱ flow cytometry system (BD Biosciences).

### Statistical analysis

Statistical analysis was performed with IBM SPSS Version 25.0 (SPSS Inc), GraphPad Prism Version 8.0 (GraphPad Prism Inc) and MedCalc Version 19.2.0 (MedCalc software). Data of normal distribution were indicated by mean ± standard deviation, and statistical comparisons between hospital admission and death were performed using Wilcoxon matched-pairs signed rank test. Correspondingly, data of abnormal distribution is expressed as median and interquartile range, comparison between 4 groups using Kruskal-Wallis test.

To explore the risk factors for disease progression from mild to more advanced types, univariable and multivariable logistic regression models were used. A bootstrap procedure was used to determine which variables would end up in the model. Twelve variables (age, gender, hypertension, diabetes, coronary heart disease, lymphocyte count, D-dimer, serum amyloid A protein, interleukin-6, procalcitonin, C-reactive protein and erythrocyte sedimentation rate) were selected for the multivariable analysis on the basis of previous findings and clinical constraints [11,12]. Previous studies have shown blood levels of D-dimer to be higher in advanced type cases, whereas lymphopenia, hypertension, diabetes and coronary heart disease have been less commonly observed in mild type patients with SARS-COV-2 infection [11]. Similar risk factors, including older age, have been reported associated with adverse clinical outcomes in adults with SARS and Middle East respiratory syndrome (MERS) [13,14].We excluded variables from the univariable analysis if their between-group differences were not significant, if the number of events was too small to calculate odds ratios. For non-normally distributed data, correlations were assessed by Spearman’s rank correlation coefficient and residuals plots. The sensitivity of different inflammatory markers and lymphocyte in the prognosis of COVID-19 patients as centrally adjudicated by two independent experts was quantified with the area.

The cumulative incidence curves (inverted Kaplan-Meier plots) with 95% confidence interval analyses were conducted using Stata version 16.0 (StataCorp). These curves examined the time from the time since COVID-19 diagnosis to the end of event (if death or curation occurs). Log-rank test was used to estimate the *P* value.

## Results

### Demographics and characteristics of patients

A total of 3,265 patients were included in this study. Their epidemiological and clinical characteristics are shown in Table S1. Based on the NHCC Guidelines, these patients at the time of confirmed diagnosis of COVID-19 were stratified as follows: mild (239 [7.3%]), ordinary (1,860 [57%]), severe (857 [26.2%]), as well as critically ill (309 [9.5%]). The median age of the cohort was 58 years (interquartile range, 46 to 67 years; range, 8 to 97 years), and 52.7% of them (1,721/3,265) were women. The most common clinical symptoms were fever (2,035 [62.3%]), cough (1,103 [33.8%]), fatigue or myalgia (695 [21.3%]), expectoration (333 [10.2%]), diarrhea (188 [5.8%]), shortness of breath (160 [4.9%]), sore throat (157 [4.8%]), as well as headache (95 [2.9%]). A proportion of patients had underlying diseases, including hypertension (487 [14.9%]), diabetes (220 [6.7%]), cerebrovascular diseases (145 [4.4%]), chronic infectious disease (96 [2.9%]), carcinoma (74 [2.3%]), chronic renal diseases (55 [1.7%]) and chronic obstructive pulmonary diseases (COPD) (30 [0.9%]). A total of 1374 patients (42.1%) were co-infected with other pathogens, such as virus (349 [10.7%]), bacteria (1,078 [33.0%]) and fungus (459 [14.1%]). An abnormal chest CT imaging was observed in 92.7% of patients. A total of 1,649 patients (50.5%) had findings of bilateral infiltrates on radiographic imaging, while 1,378 patients (42.2%) had unilateral infiltrates (Figure S1). The interval between hospital admission and discharge in survivors was 14 days (IQR, 9 to 20 days), whereas that between hospital admission and death in non-survivors was 12 days (IQR, 5 to 20 days) (Table S1).

### Laboratory parameters

Next, we determined the hematological and biochemical parameters of 3,265 COVID-19 patients. As shown in Table 1, when the disease severity gradually increased from mild type to critically ill type, patients exhibited more decreased lymphocyte and eosinophil counts, as well as decreased hemoglobin in the blood test. Moreover, significant changes in several biochemical parameters were observed, including decreased total plasma protein and albumin, as well as elevated β2 microglobulin and lactate dehydrogenase (LDH). Analysis of inflammatory profile showed that critically ill cases exhibited significantly higher levels of procalcitonin, C-reactive protein (CRP), serum amyloid A protein (SAA), erythrocyte sedimentation rate (ESR) and interleukin-6 (IL-6) than other types (all *P* < 0.001) did. The most protruding abnormality in coagulation profile was the robust elevated level of D-dimer in critically ill cases, with a median level of 1.26 μg/mL ([IQR, 0.44 to 3.63].

**Table 1.** Laboratory parameters of 3265 COVID-19 patients in hospital admission. Variables are abnormal distribution and shows by median (interquartile range). P values are calculated by Kruskal-Wallis test unless stated otherwise

Variables		Normal Range	All patients (n = 3265)	Mild (n = 239)	Ordinary (n = 1876)	Severe (n = 862)	Critically ill (n = 288)	*P*
Blood test								
White blood cell count (×10^9^/L)		3.5–9.5	5.61	5.57	5.44	5.65	7.02	< 0.001
			(4.51–7.02)	(4.63–6.93)	(4.40–6.67)	(4.54–7.02)	(4.79–10.43)	
Lymphocyte count (×10^9^/L)		1.1–3.2	1.39	1.56	1.44	1.41	0.77	< 0.001
			(0.95–1.81)	(1.30–1.91)	(1.02–1.87)	(1.02–1.81)	(0.48–1.18)	
Neutrophil count (×10^9^/L)		1.8–6.3	3.30	3.14	3.16	3.36	5.36	< 0.001
			(2.50–4.62)	(2.29–4.43)	(2.46–4.13)	(2.47–4.58)	(3.36–8.64)	
Monocyte count (×10^9^/L)		0.1–0.6	0.48	0.46	0.47	0.50	0.43	< 0.001
			(0.37–0.62)	(0.37–0.58)	(0.37–0.60)	(0.40–0.64)	(0.30–0.65)	
Monocyte (%)		3–10	8.60	8.00	8.80	9.00	6.70	< 0.001
			(6.90–10.40)	(6.70–10.10)	(7.20–10.50)	(7.35–10.70)	(4.40–8.83)	
Hemoglobin (g/L)		130–175	126.00	130.50	128.90	123.00	120.00	< 0.001
			(115.20–137.00)	(118.50–140.90)	(119.40–139.00)	(111.00–134.00)	(101.00–133.00)	
Platelet count (×10^9^/L)		125–350	212.00	208.00	213.00	223.00	177.50	< 0.001
			(169.00–261.00)	(175.00–239.00)	(172.30–260.00)	(175.00–277.50)	(127.00–245.30)	
Eosinophil count (×10^9^/L)		0.02–0.2	0.04	0.11	0.03	0.00	0.00	< 0.001
			(0.00–0.11)	(0.06–0.18)	(0.00–0.11)	(0.00–0.02)	(0.00–0.02)	
Basophil count (×10^9^/L)		0–0.06	0.02	0.03	0.02	0.01	0.01	< 0.001
			(0.01–0.03)	(0.02–0.04)	(0.01–0.03)	(0.01–0.02)	(0.01–0.03)	
Biochemical test								
Total plasma protein (g/L)		65–85	66.20	69.65	67.30	65.00	61.40	< 0.001
			(62.30–70.20)	(65.63–72.70)	(63.70–71.10)	(61.20–68.60)	(57.30–65.28)	
Globulin (g/L)		20–30	28.70	28.10	28.70	29.10	29.35	0.009
			(26.30–31.50)	(25.20–31.10)	(26.30–31.38)	(27.35–31.75)	(27.03–32.18)	
Albumin (g/L)		40–55	37.90	41.70	38.90	36.40	32.55	< 0.001
			(34.80–40.60)	(38.23–44.00)	(36.48–41.30)	(33.70–39.00)	(29.00–35.90)	
Alanine aminotransferase (U/L)		9–50	23.00	20.00	23.00	22.00	27.00	0.001
			(15.00–38.00)	(12.25–37.75)	(15.00–38.00)	(14.00–36.90)	(17.00–44.55)	
Glutamic oxaloacetic transaminase (U/L)		15–40	22.00	20.50	22.00	20.00	31.00	< 0.001
			(17.00–31.00)	(16.00–26.00)	(17.00–30.00)	(16.00–29.00)	(21.00–50.50)	
Blood urine nitrogen (mmol/L)		2.8–7.6	4.60	4.11	4.39	4.90	6.85	< 0.001
			(3.70–5.80)	(3.36–5.18)	(3.53–5.34)	(3.90–6.10)	(4.60–10.86)	
Creatinine (*μ*mol/L)		64–104	64.30	58.40	63.05	66.20	69.70	< 0.001
			(53.70–76.80)	(47.58–69.68)	(52.90–74.83)	(55.40–78.60)	(57.10–102.00)	
Uric acid (*μ*mol/L)		208–428	302.80	344.90	311.00	288.00	264.00	< 0.001
			(239.00–377.00)	(279.50–417.50)	(249.00–380.00)	(229.20–365.00)	(199.60–370.20)	
β-2 microglobulin (*μ*g/L)		1000–3000	1670.00	1461.00	1700.00	2681.00	3006.00	< 0.001
			(1376.00–2066.00)	(1277.00–1700.00)	(1411.00–2060.00)	(1793.00–3113.00)	(1955.00–5145.00)	
Lactate dehydrogenase (U/L)		125–243	187.50	154.00	179.00	198.00	299.50	< 0.001
			(160.00–234.00)	(138.00–179.00)	(155.00–212.00)	(167.60–237.00)	(225.00–436.30)	
Brain natriuretic peptide (pg/mL)		<100	47.60	19.00	30.10	84.15	63.30	< 0.001
			(13.15–107.60)	(10.00–45.08)	(10.00–77.03)	(48.98–217.80)	(20.55–205.90)	
Highly sensitive troponin I (pg/mL)		0–26.2	5.10	1.45	3.70	6.55	13.10	< 0.001
			(1.70–11.40)	(0.80–3.13)	(1.50–8.80)	(3.20–9.90)	(6.60–49.00)	
Inflammatory profile								
Procalcitonin (ng/mL)								
< 0.05^†^	< 0.05		2268 (69.5)	230 (96.2)	1416 (75.5)	528 (61.3)	94 (32.6)	< 0.001 ^‡^
> 0.05			0.13	0.11	0.08	0.08	0.17	< 0.001
			(0.07–0.28)	(0.09–0.13)	(0.06–0.14)	(0.06–0.14)	(0.09–0.56)	
C-reactive protein (mg/L)		0–10	2.91	1.50	2.70	2.83	44.40	< 0.001
			(0.92–16.73)	(0.90–3.08)	(0.90–12.49)	(0.62–16.01)	(9.37–87.53)	
hypersensitive C-reactive protein (mg/L)		0–3	7.26	1.21	6.10	27.85	75.30	< 0.001
			(1.53–43.17)	(0.77–2.02)	(1.60–32.40)	(10.45–78.73)	(34.48–137.80)	
Serum amyloid A protein (mg/L)		0–10	11.93	5.42	13.30	24.74	117.40	< 0.001
			(5.46–107.00)	(4.39–7.87)	(6.09–113.80)	(5.00–129.30)	(72.76–197.10)	
Interleukin-6 (pg/mL)		0–7	3.48	2.46	3.21	2.97	30.59	< 0.001
			(1.50–12.60)	(1.57–4.90)	(1.50–10.77)	(1.50–10.42)	(10.41–71.96)	
Erythrocyte sedimentation rate (mm/h)		0–20	20.00	8.50	20.00	24.00	38.00	< 0.001
			(9.00–35.00)	(4.25–20.00)	(9.00–34.00)	(18.00–39.75)	(20.50–56.00)	
Coagulation profile								
Prothrombin time (s)		9.4–12.5	11.50	11.50	11.40	11.40	12.50	< 0.001
			(11.00–12.30)	(10.90–12.20)	(11.00–12.30)	(11.00–12.00)	(11.70–13.60)	
Activated partial thromboplastin time (s)		21.5–36.5	29.10	30.40	29.50	27.50	29.90	< 0.001
			(26.10–32.15)	(28.45–32.70)	(26.60–32.40)	(24.40–30.50)	(27.13–33.18)	
Thrombin time (s)		10.3–16.6	16.90	14.70	16.60	17.50	16.50	< 0.001
			(15.20–17.90)	(13.90–15.50)	(14.80–17.80)	(16.70–18.40)	(15.10–17.90)	
Fibrinogen (g/L)		2–4	3.26	3.35	3.12	3.24	3.99	< 0.001
			(2.66–4.03)	(2.92–3.74)	(2.62–3.88)	(2.58–4.08)	(3.12–4.51)	
D-dimer (μg/mL)		0–0.55	0.32	0.14	0.25	0.54	1.26	< 0.001
			(0.17–0.86)	(0.08–0.40)	(0.14–0.47)	(0.25–1.23)	(0.44–3.63)	
Immune parameters								
4/8 Ratio		0.96–2.05	1.48	1.34	1.50	1.51	2.24	< 0.001
			(1.13–2.12)	(1.12–1.77)	(1.15–2.14)	(1.01–2.47)	(1.23–4.16)	
CD16^+^CD56^+^ (count/μL)		210–1514	183.00	203.00	188.00	115.00	41.50	< 0.001
			(110.00–300.00)	(142.00–403.00)	(113.00–300.00)	(73.00–228.00)	(20.25–105.00)	
CD19^+^ (count/μL)		240–1317	153.00	203.00	149.00	86.00	62.00	< 0.001
			(87.50–241.00)	(136.50–278.50)	(87.00–238.50)	(56.75–170.00)	(32.00–136.00)	
CD3^+^CD8^+^ (count/μL)		345–2350	341.00	439.00	341.00	149.50	88.00	< 0.001
			(225.50–504.00)	(328.00–607.50)	(239.50–494.00)	(82.50–542.00)	(33.50–189.50)	
CD3^+^CD4^+^ (count/μL)		345–2350	542.00	623.00	552.00	259.50	185.00	< 0.001
			(332.00–745.50)	(469.50–840.50)	(345.00–750.50)	(200.50–396.00)	(94.00–331.50)	

**^†^**Value is numbers (percentage).

**^‡^***P* value is calculated by χ2 test.

In regard to the immune parameters, as shown in Table 2, with the deterioration of the illness, patients exhibited gradually decreased CD16^+^CD56^+^NK cells, CD19^+^B cells, CD3^+^CD4^+^T cells and CD3^+^CD8^+^T cells (all *P* < 0.001) in the peripheral blood, whereas CD4^+^/CD8^+^ ratio was increased (*P* < 0.001).

**Table 2.** Risk factors for disease progression from mild to more advanced types identified by binary logistic regression analysis

☐ Variables	Univariable	*P* value	Multivariable	*P* value
	OR (95% CI)		OR (95% CI)	
Demographics and clinical characteristics				
Age, years	1.054	< 0.001	1.032	< 0.001
	(1.042–1.066)		(1.017–1.048)	
Gender	1.023	0.88		
	(0.761–1.374)			
Comorbidity present (vs not present)				
Coronary heart disease	0.905	0.88		
	(0.243–3.371)			
Diabetes	2.344	0.053	1.945	0.20
	(0.987–5.567)		(0.706–5.363)	
Hypertension	9.570	0.002	0.700	0.68
	(2.320–39.474)		(0.127–3.860)	
Laboratory findings				
Lymphocyte count (×10^9^/L)	0.301	< 0.001	1.026	0.90
	(0.230–0.393)		(0.698–1.509)	
D-dimer (μg/mL)	2.003	< 0.001	1.157	0.49
	(1.431–2.804)		(0.767–1.745)	
Serum amyloid A protein (mg/L)	1.368	< 0.001	1.303	< 0.001
	(1.276–1.466)		(1.216–1.396)	
Interleukin-6 (pg/mL)	1.379	< 0.001	1.031	0.40
	(1.273–1.495)		(0.961–1.107)	
Procalcitonin (ng/mL)	6.651	< 0.001	0.853	0.74
	(3.552–12.452)		(0.338–2.154)	
C-reactive protein (mg/L)	1.390	< 0.001	0.979	0.50
	(1.277–1.512)		(0.920–1.041)	
Erythrocyte sedimentation rate (mm/h)	1.098	< 0.001	1.025	0.010
	(1.077–1.120)		(1.006–1.045)	

### Treatments and clinical outcomes

In terms of treatments, patients mainly received antiviral, antibacterial, anti-fungal, as well as corticosteroids. 2,094 patients (64.1%) were given antiviral drugs during hospitalization, including arbidol, oseltamivir, ribavirin, interferon-α, as well as lopinavir/ritonavir. 1,510 patients (46.2%) received antibacterial therapy, including moxifloxacin, meropenem, tigecycline, as well as biapenem (Table S1). A proportion of patients (431 [13.2%]) were given anti-fungal treatment. 473 patients (14.5%) were administered with systematic corticosteroid therapy. One-fifth of patients (646 [20.0%]) were supported with high-flow oxygen. 296 (9.1%) required noninvasive ventilation and 161 patients (4.9%) needed invasive mechanical ventilation in hospitalization. Moreover, 223 patients (6.8%) were given renal replacement therapy and 13 patients (0.4%) received extracorporeal membrane oxygenation (ECMO) for rescue therapy.

The most common complications were acute respiratory distress syndrome (ARDS) (663 [20.3%]), acute cardiac injury (ACI) (508[15.6%]), acute kidney injury (AKI) (287 [8.8%]) and shock (243 [7.4%]).

Of 3,265 patients, 152 finally progressed to death, with the overall death rate of 4.7% (152/3,265). Of these non-survivors, 22.37% (34/152) were ordinary, 19.74% were severe (30/152) and 57.89% (88/152) were critically ill cases.

### The predictive value of SAA-containing parameters for the disease progression

**Figure 1.**
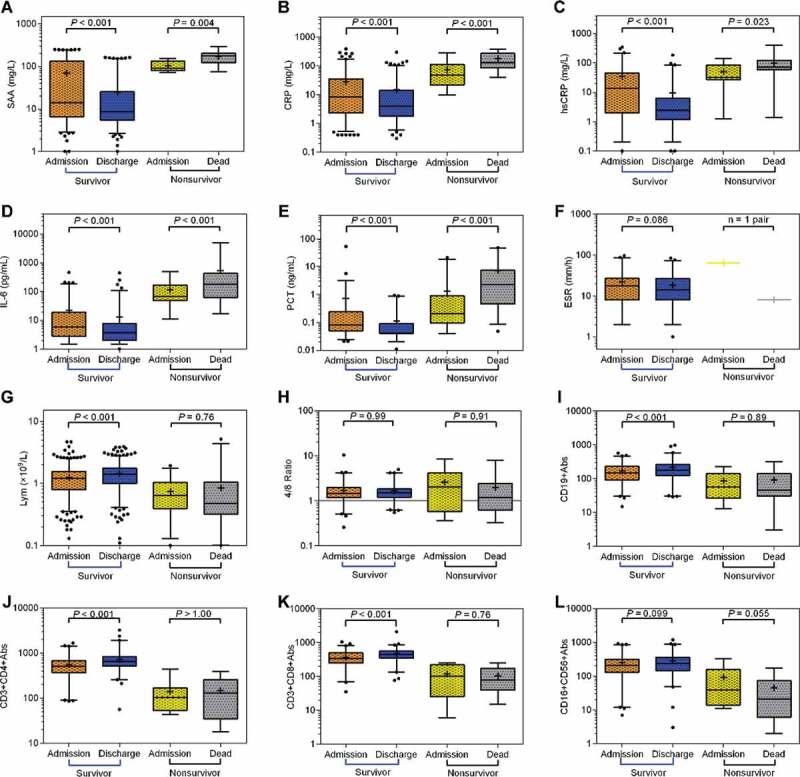
**The comparison of inflammatory and immune parameters between the date on hospital admission and hospital discharge or death in survivors and non-survivors**. The levels of the following parameters were compared at two time points in survivors and non-survivors, respectively. (a) SAA (survivors, n = 299; non-survivors, n = 9), (b) CRP (survivors, n = 289; non-survivors, n = 16), (c) hsCRP (survivors, n = 138; non-survivors, n = 17), (d) IL-6 (survivors, n = 180; non-survivors, n = 28), (e) PCT (survivors, n = 115; non-survivors, n = 60), (f) ESR (survivors, n = 84; non-survivors, n = 1), (g) lymphocyte count (survivors, n = 659; non-survivors, n = 72), (h) CD4/CD8 ratio (survivors, n = 120; non-survivors, n = 9), (i) CD19^+^B cell count (survivors, n = 120; non-survivors, n = 9), (j) CD3^+^CD4^+^T cell count (survivors, n = 120; non-survivors, n = 9), (k) CD3^+^CD8^+^T cell count (survivors, n = 120; non-survivors, n = 9), (l) CD16^+^CD56^+^NK cell count (survivors, n = 120; non-survivors, n = 9). *P* values were calculated by Wilcoxon matched-pairs signed rank test

Next, we further determined changes in several inflammatory parameters in recovered patients and non-survivors between their hospital admission and hospital discharge or death. As shown in Figure 1 and Table S2, we observed that, by comparison with the day of hospital admission, the survivors on the day of hospital discharge exhibited significantly decreased serum levels of SAA (median, 8.58 [IQR, 5.44 to 25.20] vs 14.07 [IQR, 6.55 to 133.20], *P* < 0.001), CRP (median, 4.00 [IQR, 1.8 to 14.15] vs 8.26 [IQR, 2.30 to 35.35], *P* < 0.001), hsCRP (median, 2.41 [IQR, 1.16 to 6.25] vs 13.75 [IQR, 1.96 to 45.29], *P* < 0.001), PCT (median, 0.04 [IQR, 0.04 to 0.09] vs 0.08 [IQR, 0.05 to 0.24], *P* < 0.001), as well as IL-6 (median, 3.73 [IQR, 2.00 to 7.71] vs 5.98 [IQR, 2.79 to 19.10], *P* < 0.001). In contrast, the non-survivors on the day of death showed significantly increased serum levels of SAA (median, 134.90 [IQR, 121.7 to 202.50] vs 90.92 [IQR, 79.51 to 134.90], *P* = 0.004), CRP (median, 129.60 [IQR, 84.68 to 274.10] vs 48.72 [IQR, 21.90 to 112.80], *P* < 0.001), hsCRP (median, 72.80 [IQR, 58.05 to 124.0] vs 32.50 [IQR, 26.91 to 84.00], *P* = 0.023), PCT (median, 2.31 [IQR, 0.45 to 7.38] vs 0.21 [IQR, 0.10 to 0.91], *P* < 0.001), as well as IL-6 (median, 179.10 [IQR, 62.29 to 425.40] vs 66.72 [IQR, 49.14 to 167.60], *P* < 0.001) in comparison with the day of hospital admission.

To determine the changes in immune parameters of COVID-19 patients, we compared lymphocyte, CD3^+^CD4^+^T cell, CD3^+^CD8^+^T cell, CD19^+^B cell, CD16^+^CD56^+^NK cell count and CD4^+^T/CD8^+^T ratio between the date of hospital discharge and hospital admission or death in survivors and non-survivors. In the survivors, lymphocyte, CD3^+^CD4^+^T cell, CD3^+^CD8^+^T cell and CD19^+^B cell count were significantly increased at the date of hospital discharge compared with that of hospital admission (Figure 1). In contrast, in non-survivors, the above-mentioned four immune parameters were decreased at the date of death compared with that of their hospital admission, although there was not statistical significance.

**Figure 2.**
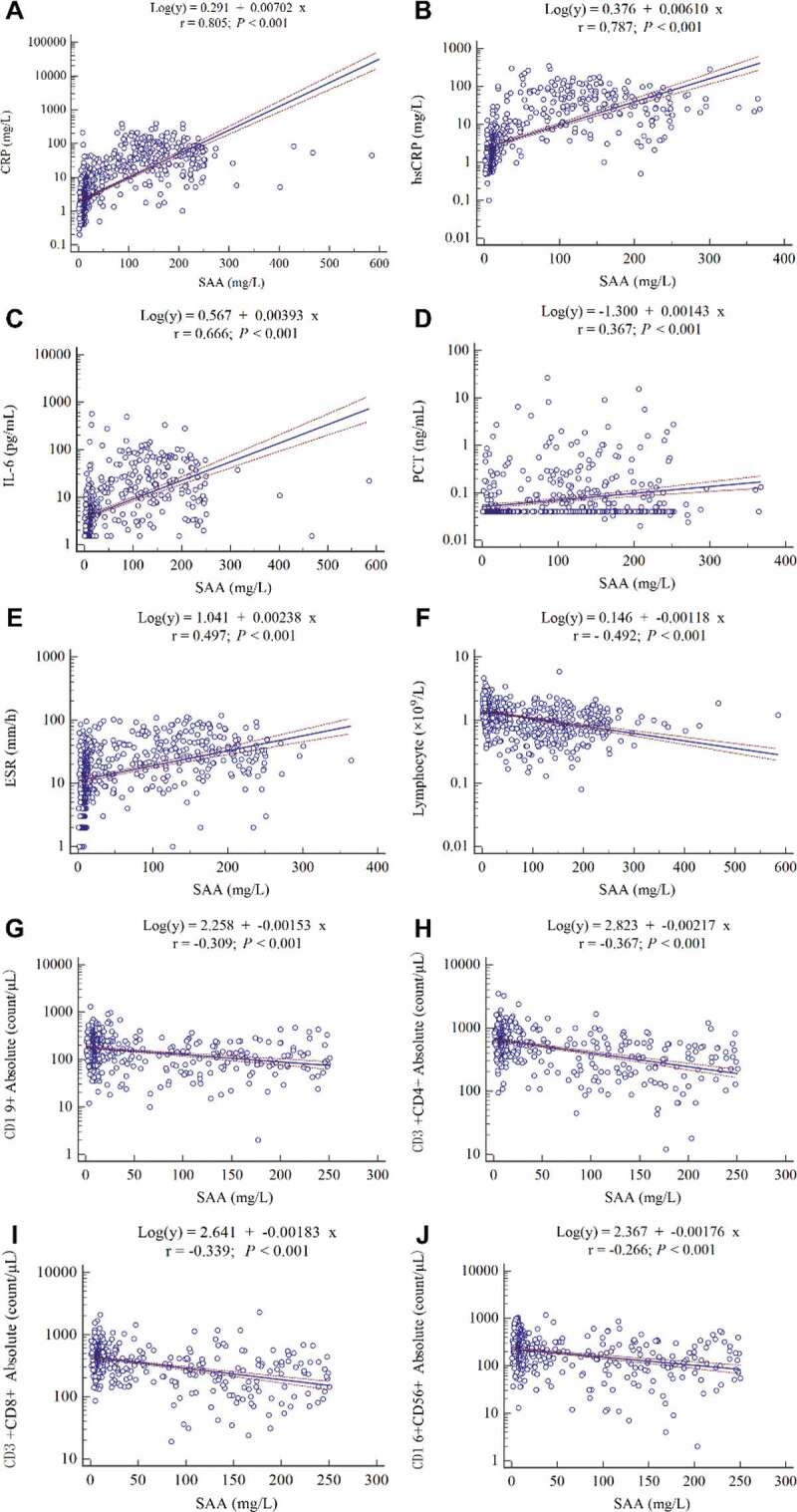
**The correlation between SAA and other laboratory parameters in COVID-19 patients**. (a) CRP (n = 946), (b) hsCRP (n = 570), (c) IL-6 (n = 581), (d) PCT (n = 580), (e) ESR (n = 677), (f) lymphocyte count (n = 857), (g) CD19^+^B cell count (n = 580), (h) CD3^+^CD4^+^T cell count (n = 580), (i) CD3^+^CD8^+^T cell count (n = 580) and (j) CD16^+^CD56^+^NK cell count (n = 580). Spearman’s correlation analysis and equation of residuals plots were shown. The dashed lines represent the 95% confidence interval of the fitted lines

The correlation analysis showed that SAA was positively correlated with CRP (r = 0.805, *P* < 0.001), hsCRP (r = 0.787, *P* < 0.001), IL-6 (r = 0.666, *P* < 0.001), PCT (r = 0.367, *P* < 0.001), as well as ESR (r = 0.497, *P* < 0.001), whereas it was negatively correlated with lymphocyte count (r = −0.492, *P* < 0.001), CD19^+^B cell count (r = −0.309, *P* < 0.001), CD3^+^CD4^+^T cell count (r = −0.367, *P* < 0.001), as well as CD3^+^CD8^+^T cell (r = −0.339, *P* < 0.001)(Figure 2(a–j)).

### The risk factors for disease progression from mild to more advanced types

Next, we used univariable and multivariable logistic regression models to determine the risk factors for disease progression from mild to more advanced types (including ordinary, severe and critically ill) of COVID-19. As shown in Table 2, univariable logistic regression model showed the following parameters had statistical significance, including age (Odds ratio,1.054 [95%CI, 1.042 to 1.066]; *P* < 0.001), hypertension (OR, 9.570 [CI, 2.320 to 39.474]; *P* = 0.002), lymphocyte count (OR, 0.301 [CI, 0.230 to 0.393]; *P* < 0.001), D-dimer (OR, 2.003 [CI, 1.431 to 2.804]; *P* < 0.001), SAA (OR, 1.368 [CI, 1.276 to 1.466]; *P* < 0.001), interleukin-6 (OR,1.379 [CI, 1.273 to 1.495]; *P* < 0.001), procalcitonin (OR,6.651 [CI, 3.552 to 12.452]; *P* < 0.001), CRP (OR,1.390 [CI, 1.277 to 1.512]; *P* < 0.001) and ESR (OR,1.098 [CI, 1.077 to 1.120]; *P* < 0.001). The multivariable logistic regression model indicated that age (OR, 1.032 [CI, 1.017 to 1.048]; *P* < 0.001), SAA (OR,1.303 [CI, 1.216 to 1.396]; *P* < 0.001) and ESR (OR,1.025 [CI, 1.006 to 1.045]; *P* < 0.001) were likely to be the risk factors for the disease progression.

### The sensitivity and specificity for SAA-containing panel for the prediction of disease progression

**Figure 3.**
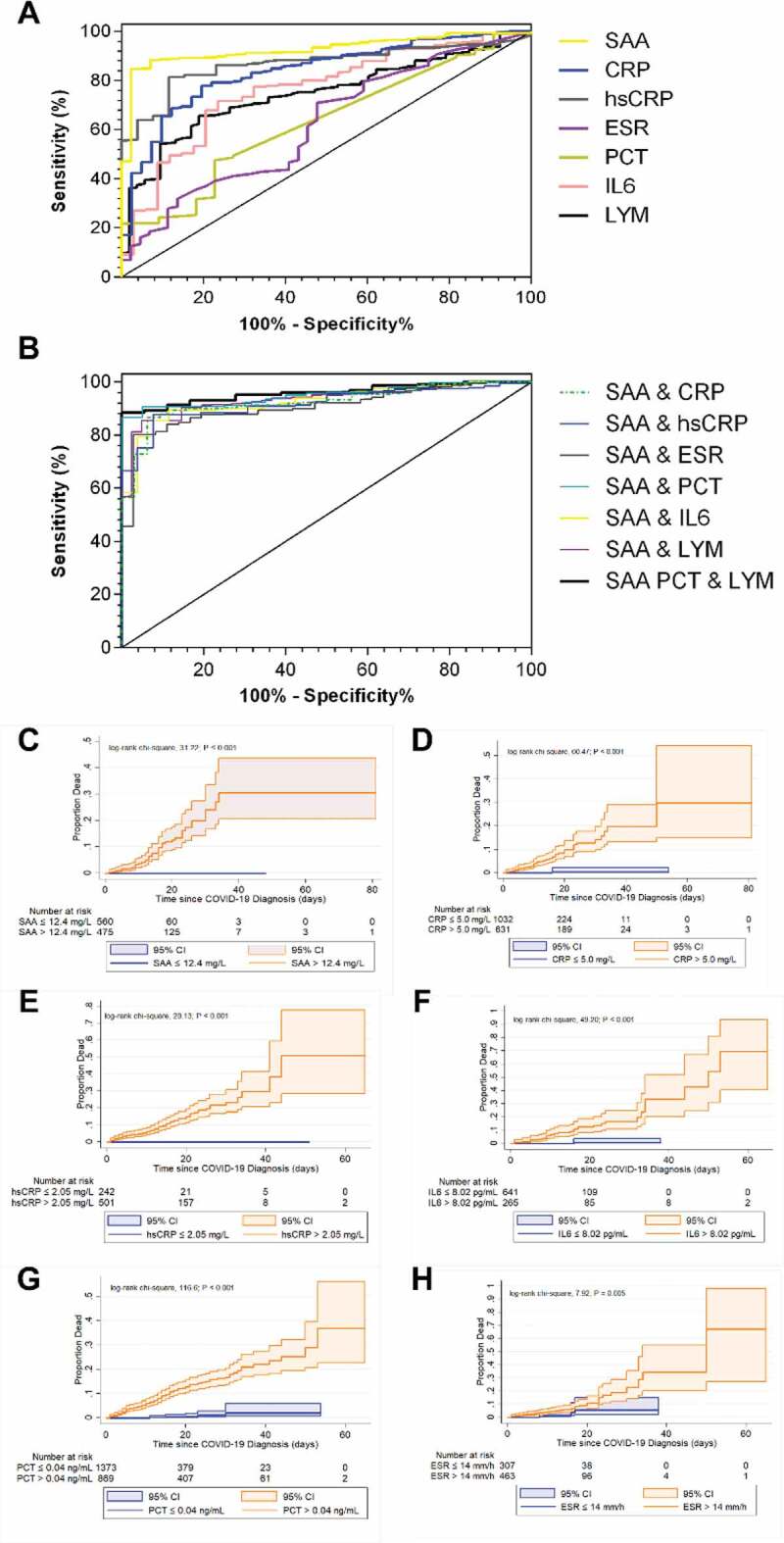
**The prediction of different bio-markers for the risk of disease progression from mild type to more advanced types and the incidence of death in different levels of markers**. (a) The areas under the ROC curves (AUC) for SAA, CRP, hsCRP, ESR, PCT, IL-6 and lymphocyte count, (b) The AUC for the various combinations. SAA plus CRP (Logit P = −0.039 + 0.056× SAA + 0.121× CRP), SAA plus hsCRP (Logit P = 0.375 + 0.035× SAA + 0.110× hsCRP), SAA plus ESR (Logit P = 0.197 + 0.075× SAA + 0.008× ESR), SAA plus PCT (Logit P = −0.453 + 0.241× SAA – 0.067× PCT), SAA plus IL-6 (Logit P = −0.113 + 0.055× SAA + 0.028× IL-6) or SAA plus lymphocyte count (Logit P = 1.837 + 0.074× SAA – 1.099× lymphocyte count). The AUC for SAA plus PCT and lymphocyte count (Logit P = 1.163 + 0.199× SAA + 0.391× PCT – 1.019× lymphocyte count). The comparison of cumulative incidence percentages in different levels of (c) SAA, (d) CRP, (e) hsCRP, (f) IL-6, (g) PCT and (h) ESR

Next, we tested the sensitivities of SAA, CRP, hsCRP, as well as IL-6 alone in the prediction of the risk of disease progression from mild to more advanced types (ordinary, severe and critically ill). As shown in Figure 3(a), the AUC from ROC analysis demonstrated that the predictive value of SAA level for the primary outcome is 0.923 (CI, 0.896 to 0.944). The best predictive cutoff value of the SAA level for the primary outcome was 12.4 mg/L, with a sensitivity of 83.9% (CI, 80.3% to 87.1%) and a specificity of 97.67% (CI, 87.7% to 99.9%). Patients with an SAA level >12.4 mg/L showed a high risk for disease progression and might need timely therapeutic intervention. The following AUCs were hsCRP (0.902) > CRP (0.885) > IL-6 (0.817) > Lymphocyte count (0.811) > ESR (0.678) > PCT (0.659) (Table 3). Moreover, the combination of SAA, PCT and lymphocyte count was identified as the most sensitive parameter for the prediction of risk of disease progression, with the AUC of 0.959 (CI, 0.934 to 0.977), the best predictive cutoff value of 0.923, a sensitivity of 88.54% (CI, 83.0% to 89.8%) and a specificity of 100% (CI, 81.5% to 100%) (Table 3). The secondary combination was SAA plus PCT, with the cutoff value of 0.923, a sensitivity of 86.67% (CI, 84.7% to 91.7%) as well as a specificity of 100% (CI, 83.2% to 100%).

**Table 3.** Cutoff values, AUC, P values, sensitivity, specificity, PLRs and NLRs for differentiation between mild type and other types of COVID-19 patients

Markers	cutoff	AUC (95%CI)	*P*	SEN (95%CI)	SPC (95%CI)	PLR (95%CI)	NLR (95%CI)
Single marker							
SAA	> 12.4 mg/L	0.923 (0.896 to 0.944)	< 0.001	83.9 (80.3 to 87.1)	97.67 (87.7 to 99.9)	36.08 (5.2 to 250.4)	0.16 (0.1 to 0.2)
CRP	> 5 mg/L	0.885 (0.845 to 0.917)	< 0.001	77.26 (72.1 to 81.9)	93.75 (79.2 to 99.2)	12.36 (3.2 to 47.4)	0.24 (0.2 to 0.3)
hsCRP	> 2.05 mg/L	0.902 (0.863 to 0.933)	< 0.001	86.18 (81.5 to 90.0)	92.31 (74.9 to 99.1)	11.2 (3.0 to 42.5)	0.15 (0.1 to 0.2)
ESR	> 14 mm/h	0.678 (0.629 to 0.725)	< 0.001	69.54 (64.4 to 74.3)	63.89 (46.2 to 79.2)	1.93 (1.2 to 3.0)	0.48 (0.4 to 0.6)
IL-6	>8.02 pg/mL	0.817 (0.760 to 0.865)	< 0.001	65.83 (58.8 to 72.4)	92.31 (74.9 to 99.1)	8.56 (2.3 to 32.5)	0.37 (0.3 to 0.5)
Lymphocyte count	≤1.27 × 10^9^/L	0.811 (0.772 to 0.846)	< 0.001	71.43 (66.8 to 75.7)	82.93 (67.9 to 92.8)	4.18 (2.1 to 8.2)	0.34 (0.3 to 0.4)
PCT	> 0.04	0.659 (0.612 to 0.704)	0.003	45.68 (40.8 to 50.7)	80 (56.3 to 94.3)	2.28 (0.9 to 5.5)	0.68 (0.5 to 0.9)
SAA combined with other markers ^†^							
SAA & CRP	> 0.76166	0.924 (0.890 to 0.950)	< 0.001	86.62 (82.2 to 90.3)	93.75 (79.2 to 99.2)	13.86 (3.6 to 53.1)	0.14 (0.1 to 0.2)
SAA & hsCRP	> 0.71861	0.919 (0.882 to 0.947)	< 0.001	87.64 (83.2 to 91.3)	92.31 (74.9 to 99.1)	11.39 (3.0 to 43.2)	0.13 (0.10 to 0.2)
SAA & ESR	> 0.799	0.908 (0.875 to 0.935)	< 0.001	80.17 (75.6 to 84.2)	97.22 (85.5 to 99.9)	28.86 (4.2 to 199.5)	0.2 (0.2 to 0.3)
SAA & IL-6	> 0.64835	0.926 (0.883 to 0.956)	< 0.001	89.45 (84.3 to 93.3)	88.46 (69.8 to 97.6)	7.75 (2.7 to 22.5)	0.12 (0.08 to 0.2)
SAA & LYM	> 0.83575	0.933 (0.906 to 0.954)	< 0.001	85.47 (81.7 to 88.7)	95.12 (83.5 to 99.4)	17.52 (4.5 to 67.7)	0.15 (0.1 to 0.2)
SAA & PCT	> 0.92335	0.95 (0.925 to 0.969)	< 0.001	86.67 (83.0 to 89.8)	100 (83.2 to 100.0)	∞	0.13 (0.1 to 0.2)
SAA & PCT & LYM	> 0.9227	0.959 (0.934 to 0.977)	< 0.001	88.54 (84.7 to 91.7)	100 (81.5 to 100.0)	∞	0.11 (0.09 to 0.2)

SAA: Serum amyloid A protein; CRP: C-reactive protein; hsCRP: hypersensitive C-reactive protein; ESR: Erythrocyte sedimentation rate; PCT: Procalcitonin; IL-6: Interleukin-6; LYM: Lymphocyte count; 95%CI: 95% confidence interval of the mean; SEN: sensitivity; SPC: specificity; PLR: positive likelihood ratio; NLR: negative likelihood ratio.

**∞**: infinite, when the positive likelihood ratio trend is infinite means the greater the probability of a true positive when the test result is positive.

**^†^** The combination of SAA with other markers used binary logistic analysis to predict a new data without specific unit of measurement.

The cumulative incidence of death was calculated from the Kaplan–Meier survival curves. Patients with SAA > 12.4 mg/L, or CRP > 5 mg/L, or hsCRP > 2.05 mg/L, or IL-6 > 8.02 pg/mL, or PCT > 0.04 ng/mL showed an increased risk for death compared with their counterparts (log rank *P* < 0.001) (Figure 3). Similarly, patients with ESR > 14 mm/h exhibited relatively higher incidence for death than those with ESR ≤ 14 mm/h (log rank *P* = 0.005).

## Discussion

It has been reported that the majority of COVID-19 patients are mild or ordinary, whereas one-fifth are severe or critically ill cases. In China, the mortality rate of COVID-19 was 2 ~ 3% [11,12,15–17]. However, in some countries, the disease mortality was over 10% [10,18,19]. At present, the urgent task for the physicians on the front lines of the pandemic is to reduce its mortality rate. Previously, we and others have demonstrated that the major deaths were derived from severe or critically ill cases [8,11,20]. A proportion of asymptomatic or cases can progress to severe or critically ill cases, which can raise the risk of death. In this regard, prevention of the disease progression from mild status to more severe stages could be a promising strategy to decrease the disease mortality. To this end, the development of biomarkers that can timely predict the risk of disease progression in patients with COVID-19 are essential.

Serum amyloid A (SAA) is an acute-phase protein during infection and inflammation [21,22]. Emerging evidence showed that SAA could be a potential marker for virus infection, such as Epstein-Barr virus [23], cytomegalovirus infection [24], hepatitis C virus [25], as well as influenza infection [26]. These aforementioned data have suggested that SAA could be an important biomarker in virus infection. An informative study has reported that a high level of SAA was observed in the serums of SARS patients [27]. Recently, Zeng et al. have demonstrated that inflammatory markers, such as SAA, CRP, PCT and ESR, were associated with the severity of COVID-19 [28]. In our study, univariable and multivariable logistic regression models have indicated that SAA, age and ESR were independent risk factors for the disease progression. Moreover, we have demonstrated that inflammatory parameters, including SAA, PCT, CRP, hsCRP and IL-6 fluctuated with the deterioration of COVID-19. Additional correlation analysis indicated that SAA was positively correlated with CRP, hsCRP, PCT, ESR, and IL-6, whereas it was negatively correlated with lymphocytes count, CD19^+^B cell, CD3^+^CD4^+^T cell and CD3^+^CD8^+^T cell count. Besides, our data is in line with other reports where patients, especially the critically ill cases, had gradually decreased lymphocyte count with disease progression [3,11]. These data suggested that decreased lymphocyte count could be a particular phenomenon in SARS-CoV-2 infection. In this regard, we determined whether the combination of SAA, PCT and lymphocyte count could be a perfect predictor for the disease progression in COVID-19. As expected, this combination achieved an AUC of 0.959, a sensitivity of 88.54% and specificity of 100%, indicating their significance as a promising predictor for disease progression. Previously, based on the data from 132 COVID-19 patients, Li et al have reported that SAA/lymphocyte count, CRP, SAA, and lymphocyte count were valuable to evaluate the disease severity [29]. Our data are consistent with this report. More importantly, our study contains a large cohort of COVID-19 patients from multiple centers thereby providing a more convincing evidence for the predictor role of SAA in the disease progression of COVID-19.

It has been well demonstrated that COVID-19 patients exhibited elevated inflammatory cytokines such as IL-1β, IL-6 and TNF-α in their serum [30]. Therefore, we hypothesized that, during the early phase of coronavirus infection with or without concomitant bacterial infection, the aforementioned cytokines are released from macrophages, which subsequently triggers the production of SAA from the cells of hepatic origin. SAA then interact with its receptors, such as TLR2, TLR4, RAGE and FPR2, and might activate the downstream signaling pathway [31]. However, the precise mechanism by which SAA plays a role in the pathogenesis of COVID-19 needs further investigation in the future.

## Supplementary Material

Supplemental Material

## Data Availability

The datasets generated and/or analysed during the current study are not publicly available due to patients’ privacy but are available from the corresponding author on reasonable request.
