# Supplementary material for: Novel biomarkers for the prediction of COVID-19 progression a retrospective, multi-center cohort study
Source: Virulence. 2020 Nov 11;11(1):1569–81. doi: 10.1080/21505594.2020.1840108 (PMC7671095; doi:10.1080/21505594.2020.1840108)
Supplement: Supplemental Material [file KVIR_A_1840108_SM9634.docx]

**Supplementary Materials**

**Supplementary Table 1. Clinical characteristics and treatments of 3265 COVID-19 patients. Values are numbers (percentage) unless stated otherwise.**

| **Variables** | **All patients (n=3265)** | |
| --- | --- | --- |
| **Median ages** (years) *^*^* | 58 (46-68) | |
| **Sex-**n (%) | | |
| Male | 1544 (47.3) | |
| Female | 1721 (52.7) | |
| **Disease severity**-n (%) | | |
| Mild | 239 (7.3) | |
| Ordinary | 1860 (57.0) | |
| Severe | 857 (26.2) | |
| Critically ill | 309 (9.5) | |
| **Underlying medical illness**-n (%) | | |
| Hypertension | 487 (14.9) | |
| Diabetes | 220 (6.7) | |
| Cerebrovascular disease | 145 (4.4) | |
| Renal disease | 55 (1.7) | |
| Carcinoma | 74 (2.3) | |
| Chronic obstructive pulmonary disease | 30 (0.9) | |
| Chronic infectious disease | 96 (2.9) | |
| **Clinical symptoms**-n (%) | | |
| Fever | 2035 (62.3) | |
| Cough | 1103 (33.8) | |
| Expectoration | 333 (10.2) | |
| Sore throat | 157 (4.8) | |
| Myalgia or fatigue | 695 (21.3) | |
| Diarrhea | 188 (5.8) | |
| Headache | 95 (2.9) | |
| Shortness of breath | 160 (4.9) | |
| Vomiting | 36 (1.1) | |
| **Bilateral involvement on chest radiographs**-n (%) | 1649 (50.5) | |
| **Unilateral involvement on chest radiographs**-n (%) | 1378 (42.2) | |
| **Interval between hospital admission and discharge** (days) *^a^* | 14 (9-20) | |
| **Interval between hospital admission to death** (days) *^a^* | 12 (5-20) | |
| **Co-infectious status**-n (%) | | |
| Virus | 349 (10.7) | |
| Bacteria | 1078 (33.0) | |
| Fungus | 459 (14.1) | |
| **Treatments**-n (%) | | |
| Antiviral therapy | 2094 (64.1) | |
| Antibiotics | 1510 (46.2) | |
| Antifugal therapy | 431 (13.2) | |
| Oxygen uptake | 646 (20.0) | |
| Corticosteroids | 473 (14.5) | |
| Non-invasive mechanical ventilation | 296 (9.1) | |
| Invasive mechanical ventilation | 161 (4.9) | |
| Renal replacement therapy | 223 (6.8) | |
| Extracorporeal membrane oxygenation (ECMO) | 13 (0.4) | |
| **Clinical outcomes** | |  |
| Alive | 3113 (95.3) | |
| Dead | 152 (4.7) | |
| **Complications** | | |
| Acute respiratory distress syndrome (ARDS) | 663 (20.3) | |
| Acute cardiac injury (ACI) | 508 (15.6) | |
| Acute kidney injury (AKI) | 287 (8.8) | |
| Shock | 243 (7.4) | |
| *^*^* Value is median (interquartile range). | | |

**Supplementary Table 2. The comparison of biochemical and immune parameters between the date of hospital admission and the date of hospital discharge or death in survivors and non-survivors with COVID-19.**

| **Parameters** | **Survivors** | | | **Non-survivors** | | |
| --- | --- | --- | --- | --- | --- | --- |
|  | **Admission** | **Discharge** | **n** | **Admission** | **Dead** | **n** |
| SAA | 14.07 | 8.58 | 299 | 90.92 | 134.90 | 9 |
|  | 6.55-133.20 | 5.44-25.20 |  | 79.51-134.90 | 121.7-202.50 |  |
| CRP | 8.26 | 4.00 | 289 | 48.72 | 129.60 | 16 |
|  | 2.30-35.35 | 1.8-14.15 |  | 21.90-112.80 | 84.68-274.10 |  |
| hsCRP | 13.75 | 2.41 | 138 | 32.50 | 72.80 | 17 |
|  | 1.96-45.29 | 1.16-6.25 |  | 26.91-84.00 | 58.05-124.0 |  |
| PCT | 0.08 | 0.04 | 115 | 0.21 | 2.31 | 59 |
|  | 0.05-0.24 | 0.04-0.09 |  | 0.10-0.91 | 0.45-7.38 |  |
| IL-6 | 5.98 | 3.73 | 180 | 66.72 | 179.10 | 28 |
|  | 2.79-19.10 | 2.00-7.71 |  | 49.14-167.60 | 62.29-425.40 |  |
| LYM | 1.20 | 1.40 | 659 | 0.64 | 0.48 | 72 |
|  | 0.79-1.56 | 1.00-1.76 |  | 0.39-1.04 | 0.32-1.05 |  |

**Fig. S1. Chest computed tomography images of a 55-year-old confirmed COVID-19 patient on day 7 after illness onset.**

Axial chest computed tomography images show ground glass opacity (**A**) in the bilateral superior lobes; (**B**) mainly in the left lower lobe; (**C**) in the superior lobe of left lung and bilateral lower lobes; (**D**) in the superior lobe of left lung, middle lobe of right lung and bilateral lower lobes.

**
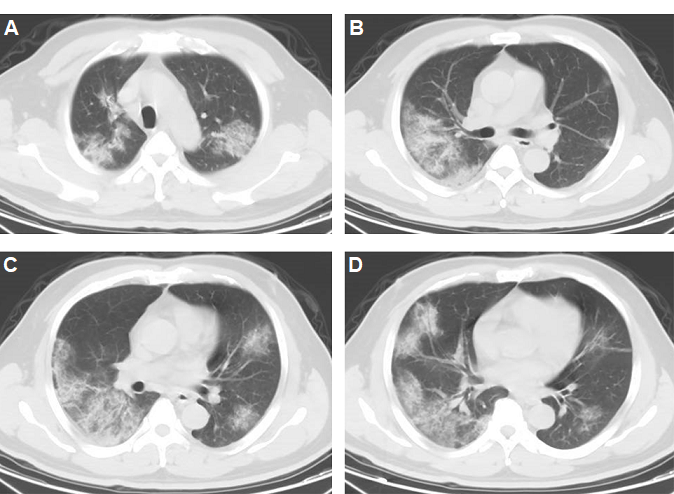
**

**Table 1.** **Laboratory parameters of 3265 COVID-19 patients in hospital admission. Variables are abnormal distribution and shows by median (interquartile range). P values are calculated by Kruskal-Wallis test unless stated otherwise.**

| **Variables** | | **Normal Range** | **All patients**  **(n = 3265)** | **Mild**  **(n = 239)** | **Ordinary**  **(n = 1876)** | **Severe**  **(n = 862)** | **Critically ill (n = 288)** | ***P*** |
| --- | --- | --- | --- | --- | --- | --- | --- | --- |
| **Blood test** | | | | | | | | |
| White blood cell count (×10^9^/L) | | 3.5-9.5 | 5.61 | 5.57 | 5.44 | 5.65 | 7.02 | < 0.001 |
|  |  |  | (4.51-7.02) | (4.63-6.93) | (4.40-6.67) | (4.54-7.02) | (4.79-10.43) |  |
| Lymphocyte count (×10^9^/L) | | 1.1-3.2 | 1.39 | 1.56 | 1.44 | 1.41 | 0.77 | < 0.001 |
|  |  |  | (0.95-1.81) | (1.30-1.91) | (1.02-1.87) | (1.02-1.81) | (0.48-1.18) |  |
| Neutrophil count (×10^9^/L) | | 1.8-6.3 | 3.30 | 3.14 | 3.16 | 3.36 | 5.36 | < 0.001 |
|  |  |  | (2.50-4.62) | (2.29-4.43) | (2.46-4.13) | (2.47-4.58) | (3.36-8.64) |  |
| Monocyte count (×10^9^/L) | | 0.1-0.6 | 0.48 | 0.46 | 0.47 | 0.50 | 0.43 | < 0.001 |
|  |  |  | (0.37-0.62) | (0.37-0.58) | (0.37-0.60) | (0.40-0.64) | (0.30-0.65) |  |
| Monocyte (%) | | 3-10 | 8.60 | 8.00 | 8.80 | 9.00 | 6.70 | < 0.001 |
|  |  |  | (6.90-10.40) | (6.70-10.10) | (7.20-10.50) | (7.35-10.70) | (4.40-8.83) |  |
| Hemoglobin (g/L) | | 130-175 | 126.00 | 130.50 | 128.90 | 123.00 | 120.00 | < 0.001 |
|  |  |  | (115.20-137.00) | (118.50-140.90) | (119.40-139.00) | (111.00-134.00) | (101.00-133.00) |  |
| Platelet count (×10^9^/L) | | 125-350 | 212.00 | 208.00 | 213.00 | 223.00 | 177.50 | < 0.001 |
|  |  |  | (169.00-261.00) | (175.00-239.00) | (172.30-260.00) | (175.00-277.50) | (127.00-245.30) |  |
| Eosinophil count (×10^9^/L) | | 0.02-0.2 | 0.04 | 0.11 | 0.03 | 0.00 | 0.00 | < 0.001 |
|  |  |  | (0.00-0.11) | (0.06-0.18) | (0.00-0.11) | (0.00-0.02) | (0.00-0.02) |  |
| Basophil count (×10^9^/L) | | 0-0.06 | 0.02 | 0.03 | 0.02 | 0.01 | 0.01 | < 0.001 |
|  |  |  | (0.01-0.03) | (0.02-0.04) | (0.01-0.03) | (0.01-0.02) | (0.01-0.03) |  |
| **Biochemical test** | | | | | | | | |
| Total plasma protein (g/L) | | 65-85 | 66.20 | 69.65 | 67.30 | 65.00 | 61.40 | < 0.001 |
|  |  |  | (62.30-70.20) | (65.63-72.70) | (63.70-71.10) | (61.20-68.60) | (57.30-65.28) |  |
| Globulin (g/L) | | 20-30 | 28.70 | 28.10 | 28.70 | 29.10 | 29.35 | 0.009 |
|  |  |  | (26.30-31.50) | (25.20-31.10) | (26.30-31.38) | (27.35-31.75) | (27.03-32.18) |  |
| Albumin (g/L) | | 40-55 | 37.90 | 41.70 | 38.90 | 36.40 | 32.55 | < 0.001 |
|  |  |  | (34.80-40.60) | (38.23-44.00) | (36.48-41.30) | (33.70-39.00) | (29.00-35.90) |  |
| Alanine aminotransferase (U/L) | | 9-50 | 23.00 | 20.00 | 23.00 | 22.00 | 27.00 | 0.001 |
|  |  |  | (15.00-38.00) | (12.25-37.75) | (15.00-38.00) | (14.00-36.90) | (17.00-44.55) |  |
| Glutamic oxaloacetic transaminase (U/L) | | 15-40 | 22.00 | 20.50 | 22.00 | 20.00 | 31.00 | < 0.001 |
|  |  |  | (17.00-31.00) | (16.00-26.00) | (17.00-30.00) | (16.00-29.00) | (21.00-50.50) |  |
| Blood urine nitrogen (mmol/L) | | 2.8-7.6 | 4.60 | 4.11 | 4.39 | 4.90 | 6.85 | < 0.001 |
|  |  |  | (3.70-5.80) | (3.36-5.18) | (3.53-5.34) | (3.90-6.10) | (4.60-10.86) |  |
| Creatinine (*μ*mol/L) | | 64-104 | 64.30 | 58.40 | 63.05 | 66.20 | 69.70 | < 0.001 |
|  |  |  | (53.70-76.80) | (47.58-69.68) | (52.90-74.83) | (55.40-78.60) | (57.10-102.00) |  |
| Uric acid (*μ*mol/L) | | 208-428 | 302.80 | 344.90 | 311.00 | 288.00 | 264.00 | < 0.001 |
|  |  |  | (239.00-377.00) | (279.50-417.50) | (249.00-380.00) | (229.20-365.00) | (199.60-370.20) |  |
| β-2 microglobulin (*μ*g/L) | | 1000-3000 | 1670.00 | 1461.00 | 1700.00 | 2681.00 | 3006.00 | < 0.001 |
|  |  |  | (1376.00-2066.00) | (1277.00-1700.00) | (1411.00-2060.00) | (1793.00-3113.00) | (1955.00-5145.00) |  |
| Lactate dehydrogenase (U/L) | | 125-243 | 187.50 | 154.00 | 179.00 | 198.00 | 299.50 | < 0.001 |
|  |  |  | (160.00-234.00) | (138.00-179.00) | (155.00-212.00) | (167.60-237.00) | (225.00-436.30) |  |
| Brain natriuretic peptide (pg/mL) | | <100 | 47.60 | 19.00 | 30.10 | 84.15 | 63.30 | < 0.001 |
|  |  |  | (13.15-107.60) | (10.00-45.08) | (10.00-77.03) | (48.98-217.80) | (20.55-205.90) |  |
| Highly sensitive troponin I (pg/mL) | | 0-26.2 | 5.10 | 1.45 | 3.70 | 6.55 | 13.10 | < 0.001 |
|  |  |  | (1.70-11.40) | (0.80-3.13) | (1.50-8.80) | (3.20-9.90) | (6.60-49.00) |  |
| **Inflammatory profile** | | | | | | | | |
| Procalcitonin (ng/mL) | | | | | | | | |
| < 0.05**^†^** | < 0.05 | | 2268 (69.5) | 230 (96.2) | 1416 (75.5) | 528 (61.3) | 94 (32.6) | < 0.001 **^‡^** |
| > 0.05 |  |  | 0.13 | 0.11 | 0.08 | 0.08 | 0.17 | < 0.001 |
|  |  |  | (0.07-0.28) | (0.09-0.13) | (0.06-0.14) | (0.06-0.14) | (0.09-0.56) |  |
| C-reactive protein (mg/L) | | 0-10 | 2.91 | 1.50 | 2.70 | 2.83 | 44.40 | < 0.001 |
|  |  |  | (0.92-16.73) | (0.90-3.08) | (0.90-12.49) | (0.62-16.01) | (9.37-87.53) |  |
| hypersensitive C-reactive protein (mg/L) | | 0-3 | 7.26 | 1.21 | 6.10 | 27.85 | 75.30 | < 0.001 |
|  |  |  | (1.53-43.17) | (0.77-2.02) | (1.60-32.40) | (10.45-78.73) | (34.48-137.80) |  |
| Serum amyloid A protein (mg/L) | | 0-10 | 11.93 | 5.42 | 13.30 | 24.74 | 117.40 | < 0.001 |
|  |  |  | (5.46-107.00) | (4.39-7.87) | (6.09-113.80) | (5.00-129.30) | (72.76-197.10) |  |
| Interleukin-6 (pg/mL) | | 0-7 | 3.48 | 2.46 | 3.21 | 2.97 | 30.59 | < 0.001 |
|  |  |  | (1.50-12.60) | (1.57-4.90) | (1.50-10.77) | (1.50-10.42) | (10.41-71.96) |  |
| Erythrocyte sedimentation rate (mm/h) | | 0-20 | 20.00 | 8.50 | 20.00 | 24.00 | 38.00 | < 0.001 |
|  |  |  | (9.00-35.00) | (4.25-20.00) | (9.00-34.00) | (18.00-39.75) | (20.50-56.00) |  |
| **Coagulation profile** | | | | | | | | |
| Prothrombin time (s) | | 9.4-12.5 | 11.50 | 11.50 | 11.40 | 11.40 | 12.50 | < 0.001 |
|  |  |  | (11.00-12.30) | (10.90-12.20) | (11.00-12.30) | (11.00-12.00) | (11.70-13.60) |  |
| Activated partial thromboplastin time (s) | | 21.5-36.5 | 29.10 | 30.40 | 29.50 | 27.50 | 29.90 | < 0.001 |
|  |  |  | (26.10-32.15) | (28.45-32.70) | (26.60-32.40) | (24.40-30.50) | (27.13-33.18) |  |
| Thrombin time (s) | | 10.3-16.6 | 16.90 | 14.70 | 16.60 | 17.50 | 16.50 | < 0.001 |
|  |  |  | (15.20-17.90) | (13.90-15.50) | (14.80-17.80) | (16.70-18.40) | (15.10-17.90) |  |
| Fibrinogen (g/L) | | 2-4 | 3.26 | 3.35 | 3.12 | 3.24 | 3.99 | < 0.001 |
|  |  |  | (2.66-4.03) | (2.92-3.74) | (2.62-3.88) | (2.58-4.08) | (3.12-4.51) |  |
| D-dimer (μg/mL) | | 0-0.55 | 0.32 | 0.14 | 0.25 | 0.54 | 1.26 | < 0.001 |
|  |  |  | (0.17-0.86) | (0.08-0.40) | (0.14-0.47) | (0.25-1.23) | (0.44-3.63) |  |
| **Immune parameters** | | | | | | | | |
| 4/8 Ratio | | 0.96 - 2.05 | 1.48 | 1.34 | 1.50 | 1.51 | 2.24 | < 0.001 |
|  |  |  | (1.13-2.12) | (1.12-1.77) | (1.15-2.14) | (1.01-2.47) | (1.23-4.16) |  |
| CD16^+^CD56^+^ (count/μL) | | 210 - 1514 | 183.00 | 203.00 | 188.00 | 115.00 | 41.50 | < 0.001 |
|  |  |  | (110.00-300.00) | (142.00-403.00) | (113.00-300.00) | (73.00-228.00) | (20.25-105.00) |  |
| CD19^+^ (count/μL) | | 240 - 1317 | 153.00 | 203.00 | 149.00 | 86.00 | 62.00 | < 0.001 |
|  |  |  | (87.50-241.00) | (136.50-278.50) | (87.00-238.50) | (56.75-170.00) | (32.00-136.00) |  |
| CD3^+^CD8^+^ (count/μL) | | 345 - 2350 | 341.00 | 439.00 | 341.00 | 149.50 | 88.00 | < 0.001 |
|  |  |  | (225.50-504.00) | (328.00-607.50) | (239.50-494.00) | (82.50-542.00) | (33.50-189.50) |  |
| CD3^+^CD4^+^ (count/μL) | | 345 - 2350 | 542.00 | 623.00 | 552.00 | 259.50 | 185.00 | < 0.001 |
|  |  |  | (332.00-745.50) | (469.50-840.50) | (345.00-750.50) | (200.50-396.00) | (94.00-331.50) |  |
| **^†^** Value is numbers (percentage).  **^‡^** *P* value is calculated by χ² test. | | | | | | | | |

**Table 2. Risk factors for disease progression from mild to more advanced types identified by binary logistic regression analysis.**

| **Variables** | **Univariable** | ***P* value** | **Multivariable** | ***P* value** |
| --- | --- | --- | --- | --- |
|  | **OR (95% CI)** |  | **OR (95% CI)** |  |
| **Demographics and clinical characteristics** | | | | |
| Age, years | 1.054 | < 0.001 | 1.032 | < 0.001 |
|  | (1.042–1.066) |  | (1.017–1.048) |  |
| Gender | 1.023 | 0.88 |  |  |
|  | (0.761–1.374) |  |  |  |
| Comorbidity present (vs not present) | | | | |
| Coronary heart disease | 0.905 | 0.88 |  |  |
|  | (0.243–3.371) |  |  |  |
| Diabetes | 2.344 | 0.053 | 1.945 | 0.20 |
|  | (0.987–5.567) |  | (0.706-5.363) |  |
| Hypertension | 9.570 | 0.002 | 0.700 | 0.68 |
|  | (2.320–39.474) |  | (0.127-3.860) |  |
| **Laboratory findings** | | | | |
| Lymphocyte count (×10^9^/L) | 0.301 | < 0.001 | 1.026 | 0.90 |
|  | (0.230–0.393) |  | (0.698-1.509) |  |
| D-dimer (μg/mL) | 2.003 | < 0.001 | 1.157 | 0.49 |
|  | (1.431–2.804) |  | (0.767-1.745) |  |
| Serum amyloid A protein (mg/L) | 1.368 | < 0.001 | 1.303 | < 0.001 |
|  | (1.276–1.466) |  | (1.216–1.396) |  |
| Interleukin-6 (pg/mL) | 1.379 | < 0.001 | 1.031 | 0.40 |
|  | (1.273–1.495) |  | (0.961-1.107) |  |
| Procalcitonin (ng/mL) | 6.651 | < 0.001 | 0.853 | 0.74 |
|  | (3.552–12.452) |  | (0.338-2.154) |  |
| C-reactive protein (mg/L) | 1.390 | < 0.001 | 0.979 | 0.50 |
|  | (1.277–1.512) |  | (0.920-1.041) |  |
| Erythrocyte sedimentation rate (mm/h) | 1.098 | < 0.001 | 1.025 | 0.010 |
|  | (1.077–1.120) |  | (1.006-1.045) |  |

**Table 3. Cut-off values, AUC, P values, sensitivity, specificity, PLRs and NLRs for differentiation between mild type and other types of COVID-19 patients.**

| **Markers** | **cut-off** | **AUC (95%CI)** | ***P*** | **SEN (95%CI)** | **SPC (95%CI)** | **PLR (95%CI)** | **NLR (95%CI)** |
| --- | --- | --- | --- | --- | --- | --- | --- |
| **Single marker** | | | | | | | |
| SAA | > 12.4 mg/L | 0.923  (0.896 to 0.944) | < 0.001 | 83.9  (80.3 to 87.1) | 97.67  (87.7 to 99.9) | 36.08  (5.2 to 250.4) | 0.16  (0.1 to 0.2) |
| CRP | > 5 mg/L | 0.885  (0.845 to 0.917) | < 0.001 | 77.26  (72.1 to 81.9) | 93.75  (79.2 to 99.2) | 12.36  (3.2 to 47.4) | 0.24  (0.2 to 0.3) |
| hsCRP | > 2.05 mg/L | 0.902  (0.863 to 0.933) | < 0.001 | 86.18  (81.5 to 90.0) | 92.31  (74.9 to 99.1) | 11.2  (3.0 to 42.5) | 0.15  (0.1 to 0.2) |
| ESR | > 14 mm/h | 0.678  (0.629 to 0.725) | < 0.001 | 69.54  (64.4 to 74.3) | 63.89  (46.2 to 79.2) | 1.93  (1.2 to 3.0) | 0.48  (0.4 to 0.6) |
| IL-6 | >8.02 pg/mL | 0.817  (0.760 to 0.865) | < 0.001 | 65.83  (58.8 to 72.4) | 92.31  (74.9 to 99.1) | 8.56  (2.3 to 32.5) | 0.37  (0.3 to 0.5) |
| Lymphocyte count | ≤1.27 ×10^9^/L | 0.811  (0.772 to 0.846) | < 0.001 | 71.43  (66.8 to 75.7) | 82.93  (67.9 to 92.8) | 4.18  (2.1 to 8.2) | 0.34  (0.3 to 0.4) |
| PCT | > 0.04 | 0.659  (0.612 to 0.704) | 0.003 | 45.68  (40.8 to 50.7) | 80  (56.3 to 94.3) | 2.28  (0.9 to 5.5) | 0.68  (0.5 to 0.9) |
| **SAA combined with other markers ^†^** | | | | | | | |
| SAA & CRP | > 0.76166 | 0.924  (0.890 to 0.950) | < 0.001 | 86.62  (82.2 to 90.3) | 93.75  (79.2 to 99.2) | 13.86  (3.6 to 53.1) | 0.14  (0.1 to 0.2) |
| SAA & hsCRP | > 0.71861 | 0.919  (0.882 to 0.947) | < 0.001 | 87.64  (83.2 to 91.3) | 92.31  (74.9 to 99.1) | 11.39  (3.0 to 43.2) | 0.13  (0.10 to 0.2) |
| SAA & ESR | > 0.799 | 0.908  (0.875 to 0.935) | < 0.001 | 80.17  (75.6 to 84.2) | 97.22  (85.5 to 99.9) | 28.86  (4.2 to 199.5) | 0.2  (0.2 to 0.3) |
| SAA & IL-6 | > 0.64835 | 0.926  (0.883 to 0.956) | < 0.001 | 89.45  (84.3 to 93.3) | 88.46  (69.8 to 97.6) | 7.75  (2.7 to 22.5) | 0.12  (0.08 to 0.2) |
| SAA & LYM | > 0.83575 | 0.933  (0.906 to 0.954) | < 0.001 | 85.47  (81.7 to 88.7) | 95.12  (83.5 to 99.4) | 17.52  (4.5 to 67.7) | 0.15  (0.1 to 0.2) |
| SAA & PCT | > 0.92335 | 0.95  (0.925 to 0.969) | < 0.001 | 86.67  (83.0 to 89.8) | 100  (83.2 to 100.0) | ∞ | 0.13  (0.1 to 0.2) |
| SAA & PCT & LYM | > 0.9227 | 0.959  (0.934 to 0.977) | < 0.001 | 88.54  (84.7 to 91.7) | 100  (81.5 to 100.0) | ∞ | 0.11  (0.09 to 0.2) |
| SAA: Serum amyloid A protein; CRP: C-reactive protein; hsCRP: hypersensitive C-reactive protein; ESR: Erythrocyte sedimentation rate; PCT: Procalcitonin; IL-6: Interleukin-6; LYM: Lymphocyte count; 95%CI: 95% confidence interval of the mean; SEN: sensitivity; SPC: specificity; PLR: positive likelihood ratio; NLR: negative likelihood ratio.  **∞**: infinite, when the positive likelihood ratio trend is infinite means the greater the probability of a true positive when the test result is positive.  **^†^** The combination of SAA with other markers used binary logistic analysis to predict a new data without specific unit of measurement. | | | | | | | |

**Figures and legends**

**Fig. 1.** **The comparison of inflammatory and immune parameters between the date on hospital admission and hospital discharge or death in survivors and non-survivors.** The levels of the following parameters were compared at two time points in survivors and non-survivors, respectively. (**A**) SAA (survivors, n=299; non-survivors, n=9), (**B**) CRP (survivors, n=289; non-survivors, n=16), (**C**) hsCRP (survivors, n=138; non-survivors, n=17), (**D**) IL-6 (survivors, n=180; non-survivors, n=28), (**E**) PCT (survivors, n=115; non-survivors, n= 60), (**F**) ESR (survivors, n=84; non-survivors, n=1), (**G**) lymphocyte count (survivors, n=659; non-survivors, n=72), (**H**) CD4/CD8 ratio (survivors, n=120; non-survivors, n=9), (**I**) CD19^+^B cell count (survivors, n=120; non-survivors, n=9), (**J**) CD3^+^CD4^+^T cell count (survivors, n=120; non-survivors, n=9), (**K**) CD3^+^CD8^+^T cell count (survivors, n=120; non-survivors, n=9), (**L**) CD16^+^CD56^+^NK cell count (survivors, n=120; non-survivors, n=9). *P* values were calculated by Wilcoxon matched-pairs signed rank test.


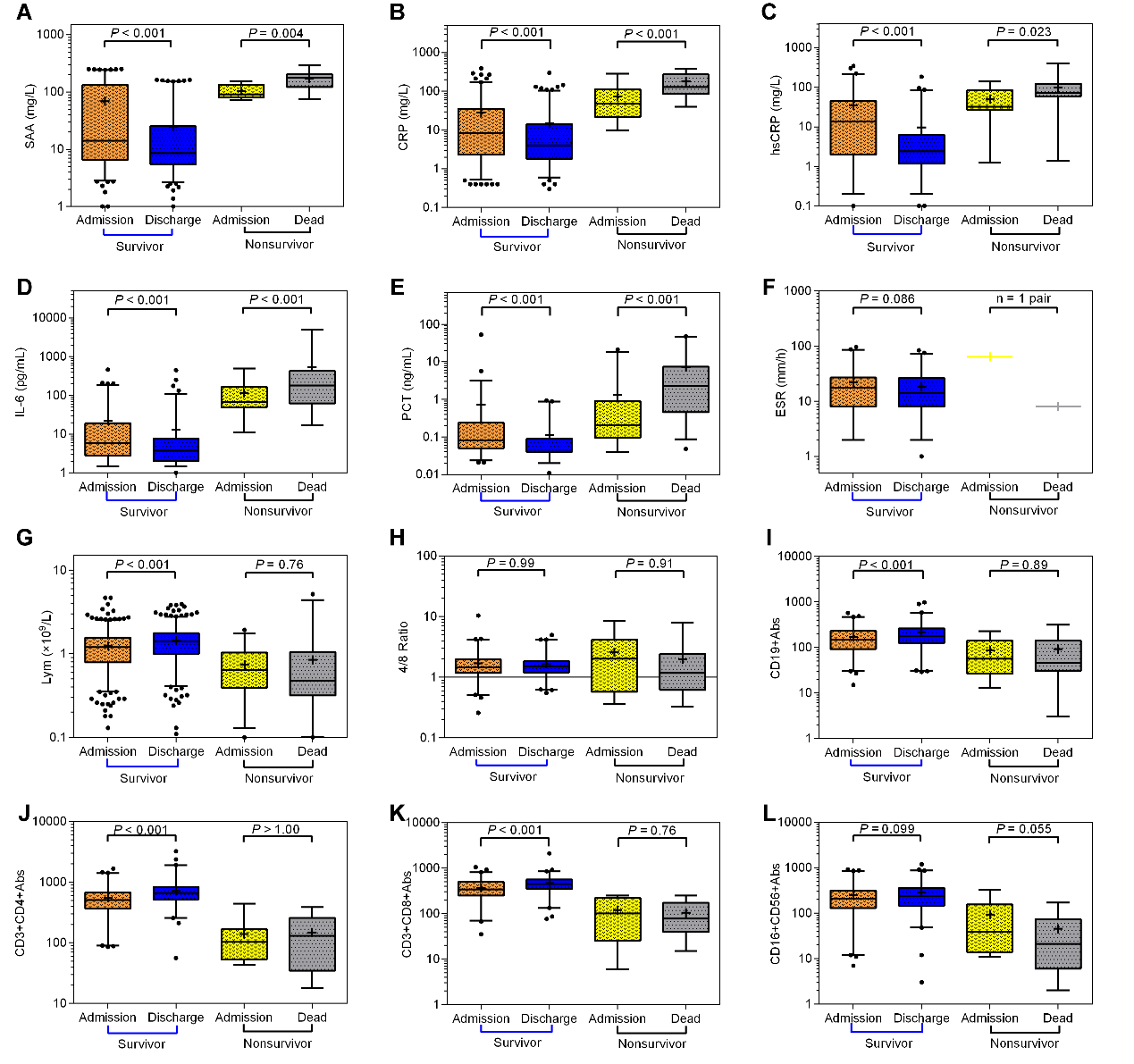


**Fig. 2.** **The correlation between SAA and other laboratory parameters in COVID-19 patients.** (**A**) CRP (n=946), (**B**) hsCRP (n=570), (**C**) IL-6 (n=581), (**D**) PCT (n=580), (**E**) ESR (n=677), (**F**) lymphocyte count (n=857), (**G**) CD19^+^B cell count (n=580), (**H**) CD3^+^CD4^+^T cell count (n=580), (**I**) CD3^+^CD8^+^T cell count (n=580) and (**J**) CD16^+^CD56^+^NK cell count (n=580). Spearman’s correlation analysis and equation of residuals plots were shown. The dashed lines represent the 95% confidence interval of the fitted lines.


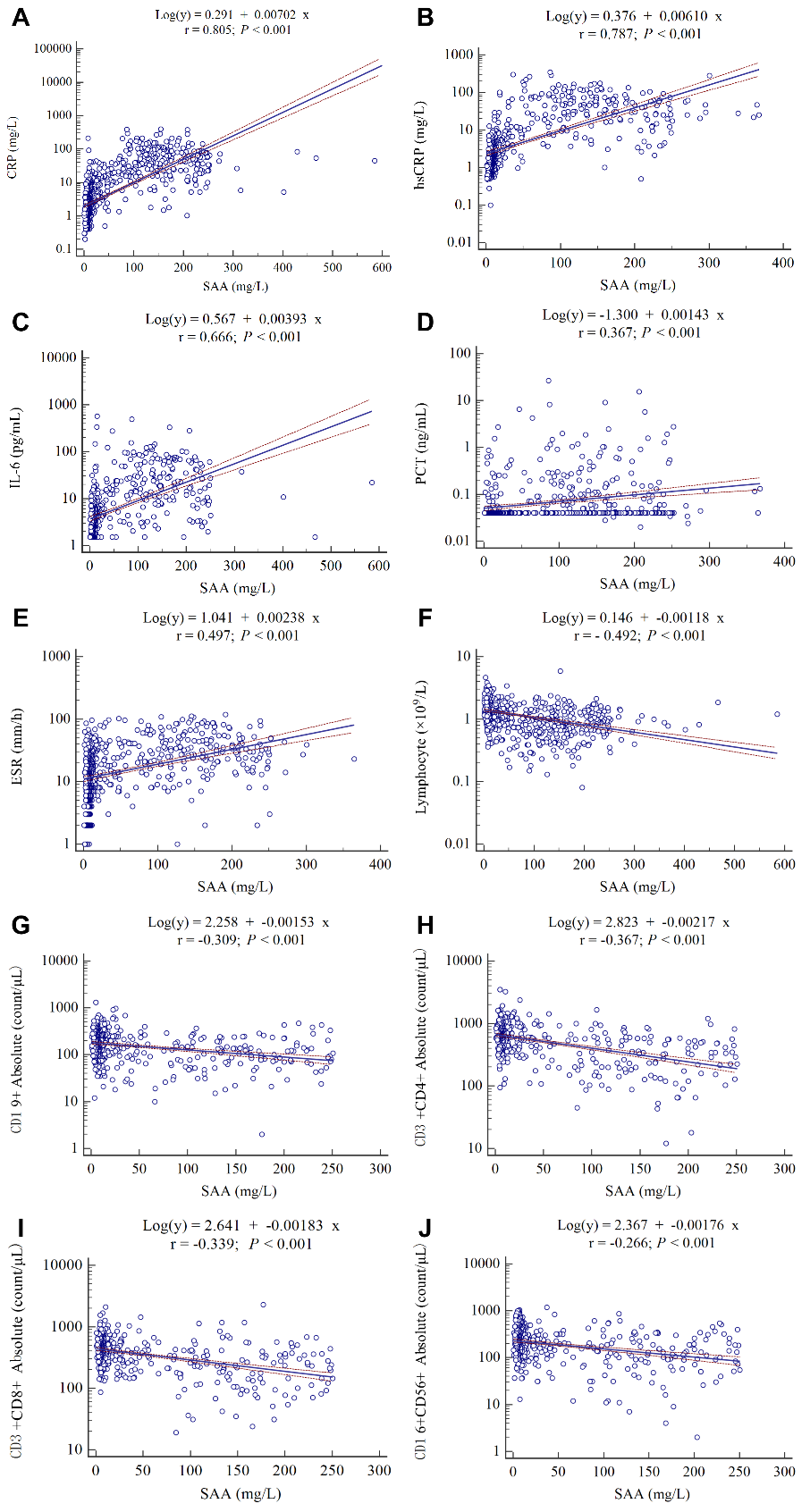


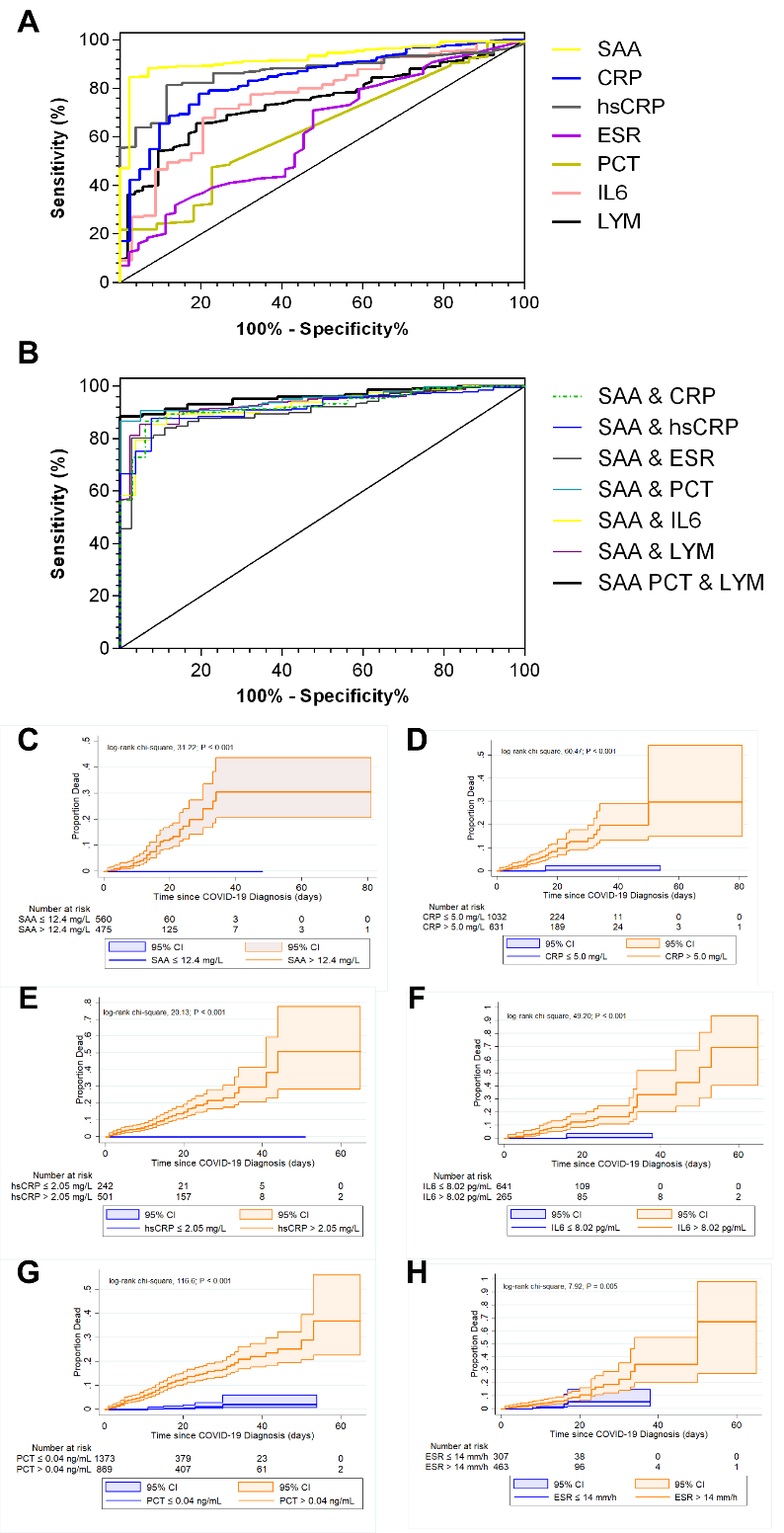
**Fig. 3.** **The prediction of different bio-markers for the risk of disease progression from mild type to more advanced types and the incidence of death in different levels of markers.** (**A**) The areas under the ROC curves (AUC) for SAA, CRP, hsCRP, ESR, PCT, IL-6 and lymphocyte count, (**B**) The AUC for the various combinations. SAA plus CRP (Logit P = -0.039 + 0.056×SAA + 0.121×CRP), SAA plus hsCRP (Logit P = 0.375 + 0.035×SAA + 0.110×hsCRP), SAA plus ESR (Logit P = 0.197 + 0.075×SAA + 0.008×ESR), SAA plus PCT (Logit P = -0.453 + 0.241×SAA - 0.067×PCT), SAA plus IL-6 (Logit P = -0.113 + 0.055×SAA + 0.028×IL-6) or SAA plus lymphocyte count (Logit P = 1.837 + 0.074×SAA - 1.099×lymphocyte count). The AUC for SAA plus PCT and lymphocyte count (Logit P = 1.163 + 0.199×SAA + 0.391×PCT - 1.019×lymphocyte count). The comparison of cumulative incidence percentages in different levels of (**C**) SAA, (**D**) CRP, (**E**) hsCRP, (**F**) IL-6, (**G**) PCT and (**H**) ESR.
